# Suppression of amyloid fibrils using the GroEL apical domain

**DOI:** 10.1038/srep31041

**Published:** 2016-08-04

**Authors:** Bimlesh Ojha, Naoya Fukui, Kunihiro Hongo, Tomohiro Mizobata, Yasushi Kawata

**Affiliations:** 1 Department of Chemistry and Biotechnology, Graduate School of Engineering Tottori University, Tottori 680-8552, Japan; 2 Department of Biomedical Science, Institute of Regenerative Medicine and Biofunction, Graduate School of Medical Science, Tottori University, Tottori 680-8552, Japan

## Abstract

In *E. coli* cells, rescue of non-native proteins and promotion of native state structure is assisted by the chaperonin GroEL. An important key to this activity lies in the structure of the apical domain of GroEL (GroEL-AD) (residue 191–376), which recognizes and binds non-native protein molecules through hydrophobic interactions. In this study, we investigated the effects of GroEL-AD on the aggregation of various client proteins (α-Synuclein, Aβ42, and GroES) that lead to the formation of distinct protein fibrils *in vitro*. We found that GroEL-AD effectively inhibited the fibril formation of these three proteins when added at concentrations above a critical threshold; the specific ratio differed for each client protein, reflecting the relative affinities. The effect of GroEL-AD in all three cases was to decrease the concentration of aggregate-forming unfolded client protein or its early intermediates in solution, thereby preventing aggregation and fibrillation. Binding affinity assays revealed some differences in the binding mechanisms of GroEL-AD toward each client. Our findings suggest a possible applicability of this minimal functioning derivative of the chaperonins (the “minichaperones”) as protein fibrillation modulators and detectors.

Cellular misfolding of proteins and their progression to stable, ordered fibrillar aggregates is closely related to a number of pathological events collectively referred to as amyloid diseases or amyloidoses. Amyloid diseases represent a family of over 25 diverse pathological conditions in humans, including neurodegenerative disorders such as Alzheimer’s disease (AD) and Parkinson’s disease (PD), as well as various metabolic and genetic syndromes such as Type II diabetes and hereditary systemic amyloidosis^1^,^2^,^3^. More than 20 different types of amyloid forming proteins or peptides have been identified so far, including α-Synuclein, amyloid beta (Aβ), and superoxide dismutase^2^,^3^,^4^,^5^. Recent studies also argue that many proteins not normally associated with disease are capable of forming amyloid fibrils under suitable experimental conditions *in vitro*^6^,^7^, and it has been suggested that almost all proteins generally have at least one structural segment that is susceptible to aggregation^8^. In spite of differences in the sequences of different amyloid forming proteins, all amyloids share a common structural motif: an ordered cross-β-sheet elongated fibrillar structure with a diameter ranging from 5 to 15 nm, formed by multiple layers of β-sheet lying in a direction parallel to the fibril axis^9^,^10^. The formation of amyloid fibrils is a multistep process involving an initial nucleation event followed by transformations through different intermediate molecular forms such as oligomers, protofibrils and fibrils^11^,^12^,^13^,^14^. Each of these intermediate species seem to display their own molecular characteristics and differences in their relative toxicity toward living cells.

Extensive studies to probe the underlying mechanism of amyloid fibril formation have been performed with a view to achieving an eventual methodology to prevent the production of cytotoxic molecular species^14^,^15^,^16^,^17^,^18^. In line with this objective, many instances have been reported where various molecular chaperones, an endogenous group of proteins known to interact specifically with proteins and prevent their aggregation, have interacted to suppress or modulate the formation of amyloids. Examples of such interactions include the Hsp104-Hsp70-Hsp40 system^19^,^20^,^21^,^22^,^23^, small heat shock proteins such as αB-crystallin^24^,^25^,^26^, and members of the chaperonin family (TriC)^27^,^28^,^29^,^30^. In each of the cases above, the respective molecular chaperone was capable of specifically recognizing, mainly through hydrophobic interactions, aggregation-prone target molecules and either suppress interactions that lead to aggregation entirely or shunt these molecules toward an alternate non-toxic form. We became curious as to whether there were any underlying common principles that ran common to all of these molecular interactions.

The bacterial chaperonin GroEL from *E. coli* is a quintessential molecular chaperonin involved in the maintenance of protein integrity in this organism^31^,^32^,^33^. Detection of aggregation-prone molecules is accomplished through the apical domain of GroEL, which spans residues 191–376 of the 548-residue GroEL monomer (Fig. 1). Bound molecules are then moved into the central cavity of the GroEL 14-mer^34^,^35^,^36^,^37^, and are segregated for a predetermined interval from other similar molecules in solution. The molecular mechanism of GroEL-facilitated folding is characterized foremost by its versatility, and previous studies have shown that GroEL is capable of assisting the folding of various proteins regardless of its original source. Recent studies have shown, in fact, that GroEL is capable of recognizing and binding to various polypeptides implicated in amyloid-related diseases; NMR studies have shown explicitly that Aβ peptide^38^ and α-Synuclein^39^ are both recognized by the apical domain of GroEL and are bound at specific sites.

A notable characteristic of *E. coli* GroEL lies in the finding that the isolated apical domain of GroEL (GroEL-AD, Fig. 1) is known to retain its structural integrity and functionality in solution. Previous studies have shown that GroEL-AD possesses a high chaperone (aggregation suppressing) activity, and that GroEL-AD must have an intrinsic chaperone activity that is not dependent on structural and functional characteristics displayed by the original GroEL oligomer. This novel chaperone activity has inspired the name “minichaperone” for this domain^40^.

By utilizing the minichaperone architecture of GroEL, it becomes possible to directly analyze the numerous interactions and dynamics that are involved in the recognition and binding of fibril-forming protein molecules to GroEL. Therefore, in this study, we first establish the effects of GroEL-AD on the aggregation of various proteins that form fibril structures, including α-Synuclein, Aβ42 and GroES, using a combination of biophysical and biochemical methods (Fig. 1). Our results confirm that GroEL-AD is capable of recognizing and binding to these unfolded client proteins and suppress fibril formation of each. Curiously, further experiments revealed various differences in the modes of binding between these three proteins and GroEL-AD. These findings are discussed in context of the myriad molecular interactions that are involved in this phenomenon.

## Results

### GroEL-AD interacts with and suppresses fibrillation of multiple proteins

We have investigated the effect of GroEL-AD on the aggregation of three client proteins (α-Synuclein, Aβ42 and GroES) using the ThT binding assay^41^. Each client protein has been confirmed to form amyloid fibrils. α-Synuclein has been implicated in the pathogenesis of PD^42^,^43^,^44^,^45^ and Aβ42 deposits are correlated with the onset of AD^46^,^47^,^48^. GroES has not been implicated in the pathogenesis of any specific diseases to date; however, preparations of GroES have been shown to form characteristic amyloid fibrils under denaturing conditions such as moderate concentrations of Gdn-HCl^49^. Interestingly, intermediate oligomeric forms of GroES that are formed during fibrillogenesis display cytotoxicity toward cultured mouse neuron cells^50^. As shown in Fig. 2a–c, all three clients, α-Synuclein, Aβ42 and GroES, formed ThT-detectable molecular species after an initial incubation period ranging from 0~6 hrs. The ThT signal in each case displayed a characteristic sigmoidal curve typical to amyloid fibril formation, involving initial formation of fibril seeds followed by fibril extension^51^,^52^. The addition of GroEL-AD to each experiment dramatically affected the aggregation profile of these client proteins in a dose-dependent manner. At sub-stoichiometric molar ratios (1:0.5 for α-Synuclein, 1:1 for Aβ42 and 1:0.5 for GroES; Fig. 2a–c) the effects of GroEL-AD addition were reflected in an increase in the initial lag phase of the transition, and a decrease in the cumulative ThT fluorescence intensity after prolonged incubation. At higher ratios of client protein to GroEL-AD (1:1 and 1:2 for α-Synuclein, 1:5 and 1:10 for Aβ42, 1:1 and 1:2 for GroES), these two effects were both strengthened. For each client protein, adding a high molar excess of GroEL-AD resulted in the almost complete suppression of fibril formation, demonstrating the strong inhibitory activity of GroEL-AD on the amyloid formation of these client proteins. In control experiments, GroEL-AD by itself showed no tendency to form ThT-responsive aggregates in any of the experimental conditions that we used (Fig. 2a–d, *black traces*). The concentration of GroEL-AD required to completely suppress ThT fluorescence increase differed for each client (α-Synuclein:GroEL-AD = 1:3, Aβ42:GroEL-AD = 1:20 and GroES:GroEL-AD = 1:4), reflecting differences in efficiency on the part of GroEL-AD toward stopping the fibril formation of these three client proteins.

### Morphology of protein fibrils formed in the presence of GroEL-AD

We next assessed the effects of GroEL-AD on the structure of resultant protein fibrils using AFM (Fig. 3). As shown in the leftmost block in Fig. 3, each client protein could form typical amyloid fibrils after prolonged incubation. Incubation of GroEL-AD under similar conditions did not lead to significant aggregation, nor to amyloid fibril formation (Fig. 3, *“GroEL-AD-only”*). The addition of GroEL-AD to each client protein in substoichiometric to stoichiometric molar ratios (Fig. 3, *center block*) interestingly failed to produce any clearly apparent changes in the morphology of these amyloid fibrils, except for a slight variation in their total observable numbers and the absence of fibrillar clusters. At higher ratios of GroEL-AD to client proteins fibrillar structures were still observable. However, shorter fibrils were more apparent in each case, and a slight decrease was seen in the total amount of fibrils visible in the experiment. Under these conditions, some small, amorphous aggregates were also observed alongside the fibrils. Finally, in the presence of excess concentrations of GroEL-AD (1:3 for α-Synuclein, 1:20 for Aβ42 and 1:4 for GroES) relative to each client protein, we observed no mature fibrils, and some small spherical aggregated structures were seen instead, which may either be amorphous aggregate forms of target protein or excess GroEL-AD (Fig. 3, *rightmost block;* compare with images of GroEL-AD only, *lowest block*). Our results seem to suggest that the participation of GroEL-AD in the fibrillation reaction generally does not cause any overt changes in the fibril morphology of the fibril-forming client protein, and rather acts to suppress the amount of fibrils that are ultimately formed by each client.

In order to characterize the effects of GroEL-AD on the morphology of protein fibrils formed by the three targets of our study in more detail, we next performed transmission electron microscopy (TEM) experiments on fibrils formed by each protein in the presence of GroEL-AD (Fig. 4). In these experiments, we also performed control experiments in which bovine serum albumin was added in place of GroEL-AD at an equivalent molar concentration (Fig. 4, *blue traces*). For each experiment, the molar concentration of GroEL-AD and BSA that was added was set to the molar concentrations used in the *green traces* shown in Fig. 2 (corresponding to molar ratios of 1:1 for α-Synuclein; 1:5 for Aβ42; and 1:1 for GroES). We note here that GroEL-AD alone, and BSA alone, failed to produce ThT-positive fluorescence signals in control experiments performed in parallel (Fig. 4, *gray and green traces*, respectively).

As shown in Fig. 4, an unexpected and interesting result was observed in each of the control experiments that we performed, which showed that addition of BSA was effective in modulating the fibril formation reaction of all three target proteins to a certain extent. However, for each target protein, GroEL-AD was more effective in fibril suppression at equivalent molar concentrations, demonstrating an effect that went beyond the presumed “non-specific” effects of BSA on fibril formation. Curiously, the effects of “non-specific” BSA addition differed for each target. In the case of Aβ42, BSA addition served to slightly decrease the overall amount of ThT-positive signal with no effects in lag time or fibrillation rate (Fig. 4a, *leftmost panel*). In contrast, for GroES, BSA served to lengthen significantly the lag time, *e. g.*, the interval required to form the initial seeds from which GroES fibrils form, with minimal effect on the rate of fibrillation (Fig. 4c, *leftmost panel*). And finally, for α-Synuclein, the effect of BSA addition acted on both the lag time and the rate of fibril formation (Fig. 4b, *leftmost panel*). This differential effect of BSA addition on the fibril forming reactions of these three target proteins may reflect differences in the specific molecular interactions that propel the fibrillation reaction of each target protein.

To probe for differences in the morphologies of fibrils formed under the various conditions shown in Fig. 4, we took samples from the end of each assay that displayed positive ThT signals and subjected them to TEM analysis. The panels displayed on the *right* of Fig. 4 summarize our results. For each target protein, we were unable to detect overt differences in the fibril morphologies between each experimental condition, save for two exceptions. The first was seen in the fibril samples of Aβ formed in the presence of BSA, where we observed that the fibrils tended to be much shorter in length than the fibrils formed by Aβ alone or Aβ in the presence of GroEL-AD. The second was seen in fibril samples formed by α-Synuclein in the presence of GroEL-AD, where the width of the fibrils seemed to be markedly thinner in the TEM images, compared to the other two conditions. Apart from these two instances, the overall shape of the fibrils seemed to be unchanged, supporting overall the results observed in AFM experiments (Fig. 3).

We next probed the effects of delayed addition of GroEL-AD during the fibril formation reaction of each client protein (Fig. 5) to probe the abilities of GroEL-AD to affect the process at various stages of the reaction. Each client protein was allowed to proceed with the fibrillation reaction for a predetermined interval (α-Synuclein; for 0, 3, 8 and 24 hr (Fig. 5a), Aβ42; for 0, 0.5, 1.5 and 8 hr (Fig. 5b), and GroES; for 0, 6, 10 and 24 hr (Fig. 5c)) before adding GroEL-AD at concentrations that were sufficient to completely suppress fibril formation as determined in Fig. 2 (3-fold molar excess for α-Synuclein, 20-fold molar excess for Aβ42, and 4-fold molar excess for GroES, respectively). In each experiment, our results indicated that the delayed addition of GroEL-AD could not reverse the process of fibril formation, but was quite successful in preventing further fibril extension. The effects of GroEL-AD addition were immediate in each data trace. Complete inhibition of fibrils could be achieved only when GroEL-AD was added at the very beginning of fibril formation, irrespective of the client protein monitored. Also, in the time frame of these experiments we could not observe a state where the client proteins were able to “escape” from the effects of GroEL-AD addition; *i.e.*, the suppressive effects of GroEL-AD addition were detected throughout the course of the fibril forming reaction. From these observations, as well as the data obtained by AFM shown in Fig. 3 and the TEM images shown in Fig. 4, we concluded that GroEL-AD acts mainly by binding to soluble monomeric unfolded client protein or the various intermediates to decrease the concentration of fibril-forming molecular species in the reaction, and does not have the ability to modify the structure of protein fibrils in a detectable manner. A similar partitioning mechanism that modulates the concentration of free protein has been reported for the effects of Hsp70 and Hsp40 on the formation of oligomeric huntingtin^53^. We note, however, that in the case of α-Synuclein, GroEL-AD may be interacting additionally to slightly alter the morphology of resultant fibrils (Fig. 4, “α*Syn* + *AD”*).

### Binding mechanisms of client proteins to GroEL-AD

In order to probe the nature of the binding interactions between GroEL-AD and various client proteins in more detail, we measured the binding affinities of GroEL-AD toward each client directly using QCM-based mass measuring as shown in Figs 6 and 7. QCM is a sensitive tool to determine intermolecular binding interactions with high precision by detecting small changes in the intrinsic frequency of a quartz crystal sensor, which is caused by changes in the mass of ligands bound to host proteins immobilized onto the sensor surface^54^,^55^. Figure 6a shows representative sensorgrams of interactions between GroEL-AD and various client proteins (concentration of injected soluble protein: 100 ng/μl). We note that the resonance frequency of the sensor decreased rapidly upon injection of each client protein, and that no significant change in resonance frequency was detected when only buffer was added to sensor with immobilized GroEL-AD (Fig. 6a, *Baseline*). A closer look at the individual sensorgrams revealed more subtle differences between the binding behavior of the three client proteins. In the case of GroES and α-Synuclein, the sensorgrams more or less displayed an exponential decrease that could be analyzed further (see below). In contrast, the sensorgrams for Aβ42 were characterized by an initial rapid change in frequency followed by a pronounced and gradual drift in the ΔF signal, which might be reflective of multivalent or non-specific binding. Upon further experimentation, the ΔF values between each session were also rather erratic in experiments involving Aβ42, compared to the other two clients. This observation, taken together with the relatively small molecular size of Aβ42 and the relatively high concentrations of GroEL-AD needed to suppress fibril formation of Aβ42 (Fig. 2), suggested that the binding interactions between these two proteins were highly dynamic and transient in nature, and not suitable (too complex) for QCM analysis.

In contrast to the experiments involving Aβ42, the binding interactions for GroEL-AD:GroES and GroEL-AD: α-Synuclein were more specific and allowed us to probe the interactions between GroEL-AD and client in more detail. Figure 6b–d summarizes the results of experiments performed on immobilized GroES titrated with various concentrations of GroEL-AD in the presence of 0.4 M Gdn-HCl. As shown in Fig. 6b, binding of GroEL-AD to immobilized GroES was dependent on the concentration of GroEL-AD added, resulting in increases in the frequency change ΔF that could be analyzed to estimate *K*_d_ (Fig. 6c). As seen in Fig. 6c, the derived |ΔF| could be fitted well to the isothermal adsorption equation to obtain *K*_d_ values of (7.0 ± 1.6) × 10^−6^ (M). Fitting the raw traces in Fig. 6b to a single exponential decay function with drift also revealed the *k*_obs_ at various [GroEL-AD], and these data were also plotted to estimate *k*_on_, *k*_off_ values. It should be noted here that we selected to omit from the analysis the *k*_obs_ values from traces obtained at the highest two GroEL-AD concentrations; due to constraints in the sampling rates of the quartz balance (1 data point/sec), these two raw traces contained relatively little information of the initial exponential decay phase, and estimates of the *k*_obs_ were correspondingly inaccurate.

The interactions between GroES and GroEL-AD in the presence of 0.4 M Gdn-HCl were most consistent with a specific 1:1 binding mechanism that was essentially irreversible. Initial analysis of the *k*_obs_ *vs* [GroEL-AD] plots (Fig. 6d) indicated that fitting of the data would result in a negative value estimation for *k*_off_, and so the data in Fig. 6d were analyzed by setting this value to zero. Estimation of the *k*_on_ under this restriction resulted in a value of *k*_on_ = 4.1 × 10^4^ (M^−1^s^−1^). Due to this constraint in the data analyses, we were unable to estimate the *K*_d_ values through estimation of the reversible kinetic rate constants as initially planned. The results from Fig. 6c however are consistent with a strong and essentially irreversible binding reaction between GroES and GroEL-AD.

In contrast, the binding reaction of α-Synuclein to immobilized GroEL-AD differed in many important aspects to the binding interactions between GroEL-AD and immobilized GroES (Fig. 7). First of all, the raw binding curves obtained from the Affinix instrument could not be fitted well to the single exponential decay reaction as recommended by the manufacturer. Upon further analysis, we found that the traces obtained at each α-Synuclein concentration were best fitted to a double exponential decay equation (Fig. 7a), which suggested that the binding of α-Synuclein to GroEL-AD was best represented by two distinct binding reactions with differing apparent rate constants. Using the sum of the amplitudes derived from analyses of the traces, we were able to estimate the *K*_d_ in Fig. 7b. As a result, we estimated the *K*_d_ to be (1.23 ± 0.31) × 10^−6^ (M). Next, we estimated the *k*_on_/k_off_ values for this binding reaction using both the faster apparent rate constant (k_obs_^fast^) and the slower rate constant (k_obs_^slow^) individually (Fig. 7c,d). Using *k*_obs_^fast^, the estimated values were *k*_on_ = 1.20 × 10^3^ (M^−1^s^−1^) and *k*_off_ = 0.25 (s^−1^) (Fig. 6c). The derived *K*_d_ from these two rates equaled *K*_d_ = 2.1 × 10^−4^ (M). Next, from the k_obs_^slow^ data we estimated the respective kinetic rate constants to be *k*_on_ = 9.2 × 10^2^ (M^−1^s^−1^), and *k*_off_ = 0.017 (s^−1^) (Fig. 7d), for a derived dissociation constant of *K*_d_ = 1.8 × 10^−5^ (M).

A notable characteristic of α-Synuclein binding to GroEL-AD revealed in these analyses was that the binding mechanism involved a significant *k*_off_ rate. In contrast to GroEL-AD:GroES binding, which was essentially a 1:1 irreversible binding reaction, the data in Fig. 7 was most consistent with a dynamic binding equilibrium of α-Synuclein to GroEL-AD, with more than one, possibly two modes of binding between these two coexisting proteins. When taken together with the results for GroES and Aβ42, our results suggest that GroEL-AD is inherently capable of utilizing multiple modes of intermolecular recognition and binding to suppress the formation of various amyloid particles *in vitro*.

## Discussion

Chaperonins protect cells by maintaining the integrity of cellular proteins from stress^56^,^57^,^58^,^59^, but their role in protecting cells from various long-term amyloidogenic disorders is not apparent. Previous studies have shown that interactions between various amyloidogenic proteins and various molecular chaperones such as Hsp104, Hsp70, and Hsp40 are possible and lead to effective suppression of amyloids^19^,^20^,^21^. In order to determine the existence of a similar role for chaperonins, we have examined the effects of GroEL-AD, the apical domain fragment of the group I chaperonin GroEL from *E. coli*, on the aggregation of multiple client proteins which form amyloid fibrils. We have shown that the presence of GroEL-AD significantly inhibits the formation of amyloid fibrils of three client proteins (α-Synuclein, Aβ42, and GroES). Experimental examples that demonstrate that a specific domain fragment from a molecular chaperone is able to control protein amyloid fibril formation are relatively scarce. However, in an earlier study performed by our group, we highlighted the effects of adding the apical domain fragment from the *Thermoplasma acidophilum* group II chaperonin (Api-Ta-cpn) on the fibril formation reaction of yeast Sup35NM^60^. In this prior study, we also found that a synthetic peptide derived from the helical protrusion region of this domain could also suppress fibril formation of Sup35NM, suggesting that the ability of Api-Ta-cpn to suppress the formation of Sup35NM fibrils involved specific structural motifs localized in a specific region of the chaperonin apical domain^60^.

In the present study, we have demonstrated that a critical concentration of GroEL-AD is required for complete inhibition of amyloid fibrils in each case. The concentration of GroEL-AD required for complete inhibition varied according to the protein monitored; relatively moderate concentrations of GroEL-AD was sufficient to suppress α-Synuclein aggregation (3-fold) and GroES aggregation (4-fold) effectively; however, much higher concentrations (20-fold molar excess) was required to achieve similar effects for Aβ42 (Fig. 2d). Below these critical concentrations, the general effect of GroEL-AD was to decrease the amount of fibril that was finally formed, most likely by limiting the concentration of free aggregation-prone protein molecules in solution. When we probed the morphology of the fibrils formed in the presence of GroEL-AD, we could not detect many overt differences in fibril morphology, and this finding seems to support this basic mechanism (Figs 3 and 4). However, there was a notable exception in the case of α-Synuclein, where we observed in TEM images fibrils that seemed to be notably thinner than the fibrils that were produced in isolation, or in the presence of an unrelated protein, BSA (Fig. 4). It may be conceivable that in the case of α-Synuclein, GroEL-AD is capable of modulating fibril morphology in addition to limiting fibril growth, and this notion agreed well with the multiple modes of binding interaction that we observed between GroEL-AD and α-Synuclein, detected through QCM experiments (Fig. 7). Perhaps the different modes of GroEL-AD binding to α-Synuclein may be responsible respectively to limit fibril elongation and modulate fibril forms. Further experiments, perhaps involving mutational analysis, will be necessary to probe this interesting facet of GroEL-AD:α-Synuclein interaction.

A notable characteristic of GroEL-AD that we uncovered in the present experiments was its rather robust ability to suppress the fibril formation of various diverse polypeptide clients, under rather diverse experimental conditions. First of all, GroEL-AD was able to bind to both relatively short (42 amino acids: Aβ42) and moderate (97 amino acids: GroES, 140 amino acids: α-Synuclein) sized polypeptide clients indiscriminately. Additionally, the structures of these clients were also slightly varied, ranging from short polypeptides (Aβ42), intrinsically disordered proteins (α-Synuclein), and natively structured oligomers that were partially denatured (GroES). Although bound by a common structural characteristic (the ability to form fibrillar aggregates under prolonged incubation), the differences in structure and chemical identity between these three clients were reflected in the specific conditions under fibrillation occurs for each client, and it is very interesting that GroEL-AD was able to bind to and control the aggregation of these clients under each individual condition. Although the analysis of these binding reactions using QCM revealed a spectrum of possible mechanisms that are responsible for this promiscuous binding of GroEL-AD to proteins, we believe that the underlying binding mechanism of GroEL-AD to these three clients might reflect a common physical principle.

**Figure 1:**
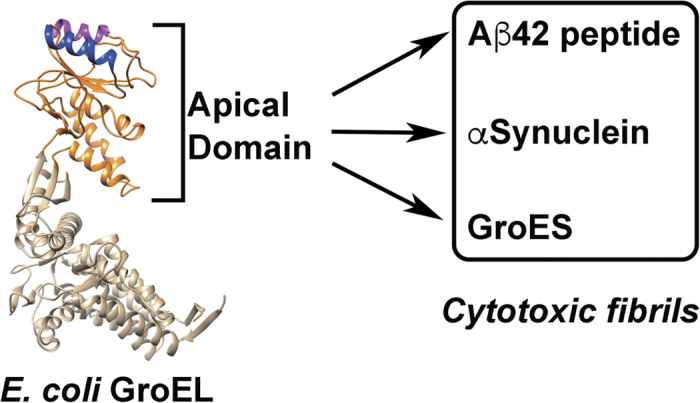
Overall concept of the present study. *Left*, structure of *E*. *coli* GroEL subunit derived from PDB 1SVT^69^. The two helical regions (Helix H, residues Leu234-Ala243 in *magenta*, and Helix I, residues Gly256-Arg268 in *blue*^70^) that form the binding interface for unfolded protein and the co-chaperonin GroES are highlighted. Models were drawn using UCSF Chimera^71^. The isolated apical domain was used to modulate the fibrillogenesis of three target peptides (Aβ42, α-Synuclein, and GroES). All three polypeptides have either been implicated in the pathogenesis of various diseases, or displayed cytotoxic tendencies in previous experiments^50^.

**Figure 2:**
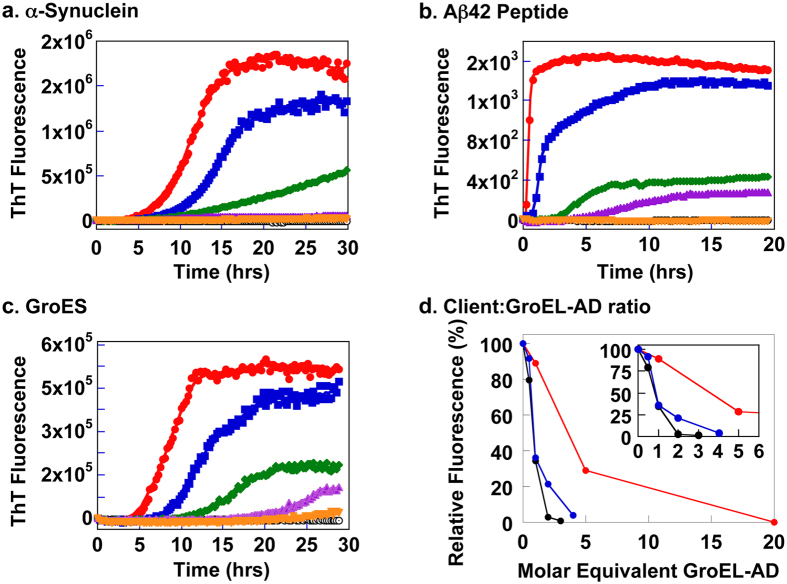
Aggregation kinetics of client proteins in the absence and presence of increasing concentrations of GroEL-AD, as accessed by ThT binding assay. (**a**) α-Synuclein; (**b**) Aβ42 peptide, (**c**) GroES. For panels (**a**–**c**), the *red filled circles* denote fluorescence values in the absence of GroEL-AD, and the *black open symbols* denote changes in ThT fluorescence caused by incubation of GroEL-AD alone under identical conditions. The concentration of GroEL-AD added to each experiment was increased according to the following progression of symbols: *blue filled squares*, *green filled diamonds*, *magenta filled triangles*, and *orange filled inverted triangles*. The specific value of client:GroEL-AD used in each sample (calculated relative to the monomeric molar concentration of client) are as follows in increasing order: (**a**) 1:0.5, 1:1, 1:2, 1:3; (**b**) 1:1, 1:5, 1:10, 1:20; (**c**) 1:0.5, 1:1, 1:2, 1:4. (**d**) Comparison of the relative effects of GroEL-AD addition on the cumulative fluorescence signal of each client protein. The values are normalized according to the fluorescence values observed for each client protein at the end of the experiment performed in the absence of additional GroEL-AD. The inset to panel (**d**) is an expansion of the main figure that shows the dependencies at low ratios of GroEL-AD to client.

**Figure 3:**
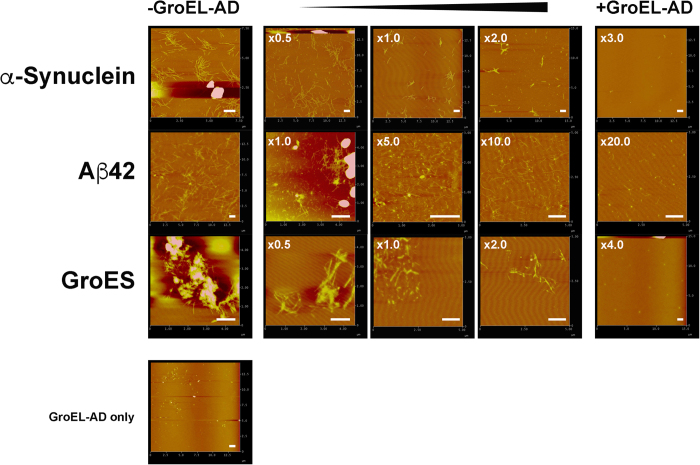
AFM images of various fibril-forming client proteins and GroEL-AD samples. Each image is a 512 × 512 pixel AFM scan of a given square area of the mica-bound sample. The *leftmost* column shows fibril samples formed in the absence of additional GroEL-AD, the three *center* columns display images of fibrils formed in the presence of increasing concentrations of additional GroEL-AD, and the *rightmost* column shows images of fibrils formed in the presence of GroEL-AD at concentrations sufficient to completely suppress the ThT fluorescence signal in assays shown in Fig. 2. *Top* (*first*) *row,* α-Synuclein, *middle* (*second*) *row*, Aβ42, *lower* (*third*) *row,* GroES. The *bottommost image* (*fourth row*) shows an image of GroEL-AD incubated under conditions identical to those used for fibril formation of α-Synuclein. Where apparent, the values at the *upper lefthand corner* of each panel denotes the actual molar equivalent of GroEL-AD that was added to samples, relative to the monomeric concentration of client protein, and at the *lower right hand corner* of each panel, a white scale bar denotes a length of 1 μm.

**Figure 4:**
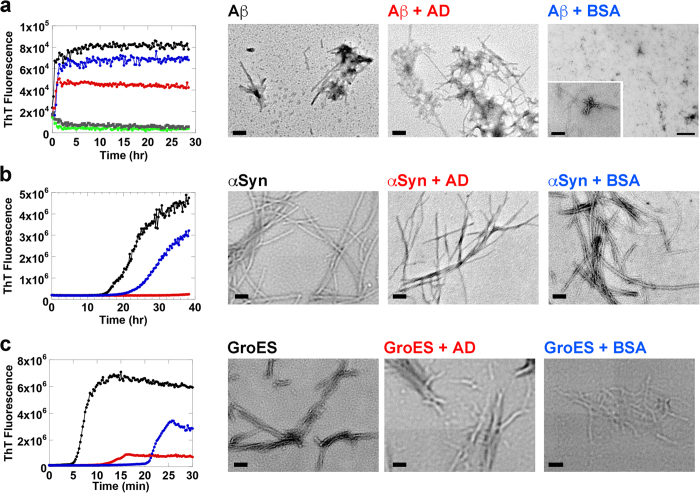
Analyses of fibril morphology using TEM. Samples of target proteins were incubated according to the conditions used in Fig. 2 and monitored with agitation in an ARVO X4 plate reader. Block a (*upper panels*) represents experiments performed on Aβ42, block b (*center panels*) represents experiments performed on α-Synuclein, and block c (*lower panels*) represents experiments involving GroES. *Gray traces* and *light green trace*s in the time trace of block a (*uppermost left*) denote changes in ThT fluorescence for BSA and GroEL-AD, respectively, at a molar concentration of 50 μM. Target proteins were either incubated alone (denoted in *black*) or in the presence of either GroEL-AD (denoted in *red*) or BSA (denoted in *blue*). The concentrations of GroEL-AD and BSA added were set to the following molar ratios relative to target monomer: Aβ42, 1:5; α-Synuclein, 1:1; and GroES, 1:1. After each experimental session, aliquots from each sample that displayed a positive ThT fluorescence signal were subject to TEM analysis. The images shown to the *right* of each time course display the results of TEM analysis. The magnification used in each panel was set to 30,000 magnification, with the exception of the “Aβ + BSA” sample panel shown in the *uppermost right corner* of the figure. In this panel the magnification is set to 4,000x magnification, and the *lower left inset* depicts an image taken at 100,000x magnification that was adjusted digitally to correspond to 30,000x magnification using image manipulation tools.

**Figure 5:**
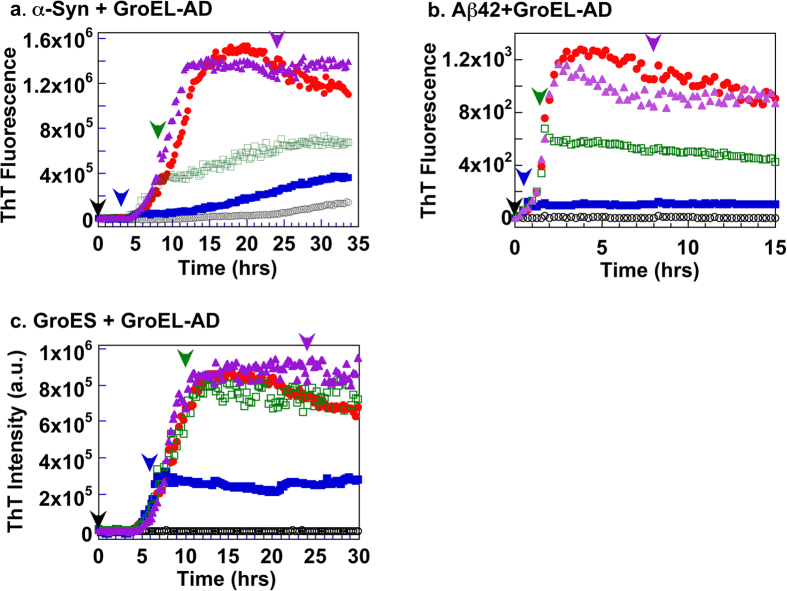
Delayed addition of GroEL-AD to the fibril forming reactions of each client protein. In each panel, *colored arrowheads* denote the instant at which excess GroEL-AD was added to each corresponding color-coded trace of the experiment. (**a**) α-Synuclein. GroEL-AD (3-fold molar excess) was added at 0 (*black*), 3 (*blue*), 8 (*green*) and 24 (*magenta*) hours after initiating the experiment. (**b**) Aβ42 peptide. GroEL-AD (20-fold molar excess) was added at 0 (*black*), 0.5 (*blue*), 1.5 (*green*) and 8 (*magenta*) hours after initiating the experiment. (**c**) GroES in 0.4 M Gdn-HCl. GroEL-AD (4-fold molar excess) was added at 0 (*black*), 6 (*blue*), 10 (*green*) and 24 (*magenta*) hours after initiating the experiment.

It is well known that GroEL senses the hydrophobicity of transiently unfolded protein molecules as they accumulate in the cell as a response to stress. It is also well established that the apical domain of GroEL is the domain which acts as the hydrophobic sensor that distinguishes and binds to these molecules (Fig. 1, helices H and I). Our experiments therefore highlight the contribution of the hydrophobic effect on the fibrillation of the three target proteins that we studied here. In a fortuitous discovery, the role of hydrophobicity in protein fibrillation was also highlighted in control experiments that we performed using BSA (Fig. 4). BSA, an unrelated serum protein that adsorbs lipids and various nutrient molecules for transport through the bloodstream, was found to affect significantly the course of fibril formation of all three polypeptides that we tested, Aβ42, α-Synuclein, and GroES, albeit in each case to a lesser extent than GroEL-AD in equivalent concentrations (Fig. 4). The interesting finding that we observed in these “control” experiments was that BSA affected the fibrillation process in a different manner for each target polypeptide, ranging from specifically lengthening the initial nucleation lag time (GroES) to altering significantly the morphology of resulting fibrils (Aβ42). The results shown here regarding the effects of BSA addition to protein fibrillation served serendipitously to highlight the many facets in which hydrophobic interactions are involved in the nucleation and extension of protein fibrils. Also, it should be mentioned here that protein fibrillation is by no means modulated exclusively by hydrophobic interactions, as previous studies have highlighted the contribution of electrostatic interactions on protein fibrillation, using various positively and negatively charged compounds on Aβ40 fibrillogenesis^61^,^62^. Protein fibrillation most likely involves numerous diverse interactions that interact spatially along the polypeptide chain, as well as through various molecular interactions that are sensitive to environmental stimuli. This idea is all the more relevant in analyses of the modulation of fibrillation through protein-protein interactions, as we are attempting here. In the present study, we believe that we have been successful in establishing a baseline from which we may probe further the numerous molecular interactions and events that underlie protein fibrillation, and intend to extend our efforts to probe common principles that underlie this important phenomenon.

## Methods

### Materials

All chemical reagents were obtained from commercial suppliers and used without further purification unless otherwise stated.

### Expression and Purification of Proteins

The gene fragment corresponding to GroEL-AD was prepared by polymerase chain reaction (PCR) amplification using pUCESL (plasmid containing the wild-type *groESL* gene) as template, and two primers that flank the apical domain sequence (5′-AGGAGATATACATATGGAAGGTATGCAGTTCGACCGT-3′ (forward) and 5′-GAATTCGGATCCGCGTTAAACGCCGCCTGCCAGT-3′ (reverse)). The PCR product was ligated into pET23a(+) vector (Novagen) and the resultant plasmid (pET-AD) was used to transform *E. coli* BLR(DE3) (Novagen). BLR(DE3)/pET-AD cells were suspended in purification buffer (50 mM Tris-HCl, pH 7.5 containing 2 mM EDTA, 2 mM DTT and 0.1 mM PMSF) followed by disruption using sonication and centrifuged. To the supernatant, streptomycin sulfate (2.5% final concentration) was added to precipitate the nucleic acids. After removal of nucleic acids by centrifugation, the supernatant was heated at 70–75 °C for 10 min, rapidly cooled on ice, and centrifuged to remove precipitated proteins. GroEL-AD protein was precipitated from this supernatant by adding fine solid ammonium sulfate to 65% saturation, centrifugation, and re-solubilization of the protein pellet in buffer. This concentrated protein solution was then loaded to a column (660 cm^3^) filled with Sephacryl S-300 (GE Healthcare) size-exclusion chromatography resin equilibrated with buffer (50 mM Tris-HCl containing 0.1 mM EDTA, 0.1 mM DTT and 100 mM NaCl; pH 7.5) and the column was developed at a flow rate of ~0.5 mL/min. Eluted samples were analyzed by SDS-PAGE and fractions containing GroEL-AD were desalted by dialysis against 5 mM sodium bicarbonate overnight, followed by dialysis against 1 mM sodium bicarbonate for 2 hr at 4 °C. The desalted protein solution was then lyophilized and stored at 4 °C. Concentrations were estimated by using a molar extinction coefficient of 4470 M^−1^cm^−1^ 63 at 280 nm for GroEL-AD.

α-Synuclein was purified as described previously from BLR(DE3) cells containing an overexpressing plasmid^64^. The concentration of α-Synuclein was estimated using a relative absorption coefficient of ε^0.1%^_280_ = 0.354^64^.

Synthetic Aβ42 peptide was purchased from Peptide Institute Inc., Japan. A working solution of 500 μM Aβ42 was prepared by dissolving ~0.42 mg of lyophilized peptide in 200 μL of 0.02% ammonium solution in a 1.5 mL eppendorf tube and kept on ice before use.

GroES was purified as described previously^65^,^66^. Purified samples were subjected to dialysis in Milli-Q water, lyophilized, and stored at 4 °C. The purity of the protein sample was checked by SDS-PAGE. The concentration of GroES solutions was determined by protein dye assay (Bio-Rad Laboratories) using bovine serum albumin (Sigma) as a standard reference.

### Aggregation Kinetics of Client Proteins Monitored by Thioflavin T (ThT) Binding Assay

The aggregation kinetics of α-Synuclein were measured as described previously using ThT^67^, an environmentally sensitive fluorophore for selective binding of amyloid fibrils^41^. Briefly, the concentrated α-Synuclein sample solution was diluted to a final concentration of 1 mg/mL in 25 mM Tris-HCl buffer, pH 7.5, containing 20 μM ThT and 150 mM NaCl. The solution was then transferred into 96-well microplate wells (Costar black, clear bottom; Greiner, Kremsmuenster, Austria), sealed using 3 inch crystal clear sealing tape (Hampton Research) and plates were loaded onto a Perkin Elmer multilabel fluorescence plate reader (ARVO X4 (VICTOR™ X), Waltham, MA, USA), where it was incubated under orbital shaking at 37 °C. The fluorescence (excitation at 450 nm, emission detected through a 486 nm/10 nm bandpass filter) was measured from the bottom of the plate at 15 min intervals, with 12 min of orbital shaking applied before each reading. Three independent experiments were performed for each set.

For monitoring the formation of GroES fibrils, concentrated sample solutions of GroES were diluted to a concentration of 1 mg/mL with 50 mM phosphate buffer, pH 7.4, containing 0.4 M guanidine hydrochloride (Gdn-HCl) and 20 μM ThT. Gdn-HCl is necessary to partially unfold GroES and promote fibril formation^49^. The concentration of Gdn-HCl used here, however, is lower than the concentration used in the previous study to characterize GroES fibril formation (0.9–1.6 M Gdn-HCl^49^); this change in denaturant concentration was necessary to prevent denaturation of the GroEL-AD fragment during experiments. The sample solution was then transferred to 96-well microplate wells that were sealed and loaded onto the ARVO X4 fluorescence plate reader at 37 °C. The fluorescence was measured in a same manner as described in the previous section for α-Synuclein.

The monomeric Aβ42 peptide solution was diluted to a final concentration of 10 μM with 50 mM phosphate buffer, pH 7.4, containing 150 mM NaCl and 20 μM ThT. One hundred fifty microliters of sample was transferred into wells of a 96-well microplate (Costar black, clear bottom), sealed and loaded onto a Gemini SpectraMax EM fluorescence plate reader (Molecular Devices, Sunnyvale, CA), and incubated at 37 °C. The fluorescence (excitation at 440 nm, emission at 485 nm) was measured from the bottom of the plate at 15 min intervals, with 5 sec of agitation before each reading. Three independent experiments were performed for each set.

Working solutions of GroEL-AD were either prepared in 25 mM Tris-HCl buffer (pH 7.5) for experiments using α-Synuclein, or in 50 mM phosphate buffer (pH 7.4) for experiments with Aβ42 and GroES. Lyophilized protein stocks were dissolved in their respective buffers and a designated concentration of GroEL-AD was added to α-Synuclein (1 mg/mL) in 25 mM Tris-HCl buffer (pH 7.5), containing 150 mM NaCl and 20 μM ThT, Aβ42 (10 μM) in 50 mM phosphate buffer (pH 7.4), containing 150 mM NaCl and 20 μM ThT, and GroES (1 mg/mL) solution in 50 mM phosphate buffer (pH 7.4), containing 0.4 M Gdn-HCl and 20 μM ThT. Each solution was then mixed briefly for 5 sec and pipetted into microplates (150 μL/well) for assays to quantitate ThT fluorescence.

Raw data from fluorescence assays were visualized using KaleidaGraph version 4.5.1 (Synergy Software, PA, USA).

### Atomic Force Microscopy (AFM)

AFM measurements were performed on a Digital Instruments Nanoscope IV scanning microscope (MMAFM-2) at room temperature using tapping mode in air. Incubated samples (α-Synuclein after 40 hr, Aβ42 after 30 hr and GroES after 40 hr respectively with and without added GroEL-AD) were diluted 10-fold and were placed on freshly cleaved mica for 30 min, washed with 100 μL of water and dried overnight at room temperature prior to imaging.

### Probing Fibril Morphology Using Transmission Electron Microscopy (TEM)

Reaction mixtures of Aβ42, α-Synuclein, and GroES were prepared as outlined above in the presence or absence of GroEL-AD or BSA and fibrillation was allowed to proceed in an ARVO X4 plate reader with agitation. The molar concentrations of GroEL-AD or BSA added corresponded to the following molar ratios relative to target monomer: Aβ42, 1:5; α-Synuclein, 1:1; and GroES, 1:1. The ThT fluorescence of each sample was monitored at regular intervals to obtain the *leftmost* traces shown in Fig. 4. After the assay was completed, aliquots were taken from each sample that displayed a positive ThT signal and used to prepare samples for TEM analysis. Ten microliters of sample were applied to carbon-coated 400-mesh copper grids (Nisshin-EM, Tokyo) and incubated for 1 min at room temperature. Sample solutions were then blotted off the grids and 5 μl Milli-Q water was added to rinse the surface. Immediately after blotting off the water rinse, 5 μl of EM-Stainer solution (a gadolinium triacetate based electron microscopy stain, Nisshin-EM, Tokyo^68^) was applied for 1 min, after which the carbon grid was again rinsed with 5 μl Milli-Q water. Grids were dried for 1 hr at room temperature before TEM analysis on a JEOL JEM-1400plus transmission electron microscope at 80 kV (Fig. 4, *right traces*).

When preparing samples of GroES fibrils formed in the presence of 0.4 M Gdn-HCl, we found that the denaturant was preventing the efficient adsorption of sample to the carbon-coated grids. Therefore, to remove denaturant prior to sample preparation, aliquots containing Gdn-HCl were first diluted tenfold with 50 mM phosphate buffer (pH 7.4), centrifuged at 15,000× g for 10 min at 4 °C, and the precipitate was resuspended in 30 μl phosphate buffer for use in the above preparations.

### Binding Interactions Between GroEL-AD and Client Proteins

The binding interactions between GroEL-AD and fibril forming client proteins were directly monitored by quartz crystal microbalance (QCM) binding analysis using a Ulvac AffinixQNμ device equipped with a 27 MHz AT-cut gold coated QCM^54^ onto which various proteins could be affixed for affinity analysis. Prior to immobilization of protein (either GroEL-AD, or GroES) to the sensor, the gold surface was cleaned with 1% SDS, followed by incubation with piranha solution (H_2_SO_4_:H_2_O_2_ = 3:1) for 5 min, and a final thorough wash with double-distilled water. In binding experiments involving α-Synuclein and Aβ42, GroEL-AD (100 ng/μL) was immobilized onto the cleaned sensor cell using protocols recommended by the manufacturer, followed by the immersion of the sensor in 0.5 mL reaction buffer (50 mM Tris-HCl, pH 7.5, containing 2 mM EDTA and 2 mM DTT). After stabilization of the basal quartz oscillation, a 5 μl aliquot of guest protein solution (α-Synuclein or Aβ42) was injected into the buffer filled cuvette to analyze the interaction between host and guest on the gold electrode.

For analysis of the interactions between GroEL-AD and GroES, in order to simulate the conditions under which GroES fibrils are formed in our experiments, we added 0.4 M Gdn-HCl to all of our QCM binding experiments involving these two proteins. Perhaps due to this change, when we initially performed experiments with GroEL-AD bound to the sensor chip and GroES as ligand, we could not detect any meaningful traces for analysis. Reversing the relationship (GroES bound to the sensor, GroEL-AD added as ligand) allowed us to obtain reliable data for analysis in the presence of 0.4 M Gdn-HCl.

For each experiment, interactions were detected by the frequency changes (oscillation unit, OU: -ΔF in Hz) caused by changes in mass bound to the electrode surface at the sub-nanogram level, attributed to specific ligand protein binding^55^. All experiments were carried out at 25 ± 1 °C with constant stirring at 1000 rpm. Between each session, the sensor with immobilized protein was incubated for 30 min with reaction buffer containing 1.6 M Gdn-HCl to remove bound guest protein, then incubated for 30 min with reaction buffer without denaturant to allow regeneration (refolding) of the immobilized protein, and finally adjusted to the conditions of each experiment. We found that this regeneration protocol, instead of using an alternative protocol involving the thorough removal and subsequent fresh immobilization of protein, tended to yield more consistent and reproducible data. In experiments involving GroES, an additional pre-incubation interval of 30 min in buffer containing 0.4 M Gdn-HCl was incorporated prior to measurements. Raw sensorgrams were either fitted to a single exponential decay equation corrected for drift to elucidate apparent rate constants (*k*_obs_) and the net change in oscillation frequency (ΔF), or alternatively, fitted to a double exponential decay equation to obtain ΔF and two apparent rate constants, fast (*k*_obs_^fast^) and slow (*k*_obs_^slow^). Estimation of the dissociation constant (*K*_d_) between GroEL-AD and each client were estimated using two different methods; in the kinetic estimation method, the rates of ligand binding (*k*_on_) and ligand dissociation (*k*_off_) were estimated from linear regression analysis of the *k*_obs_ against [Client], according to the following equation:


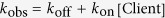


Alternatively, the *K*_d_ was estimated directly from non-linear fitting of plots of the |ΔF| against [Client] according to the Langmuir equation for isothermal adsorption:


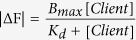


**Figure 6:**
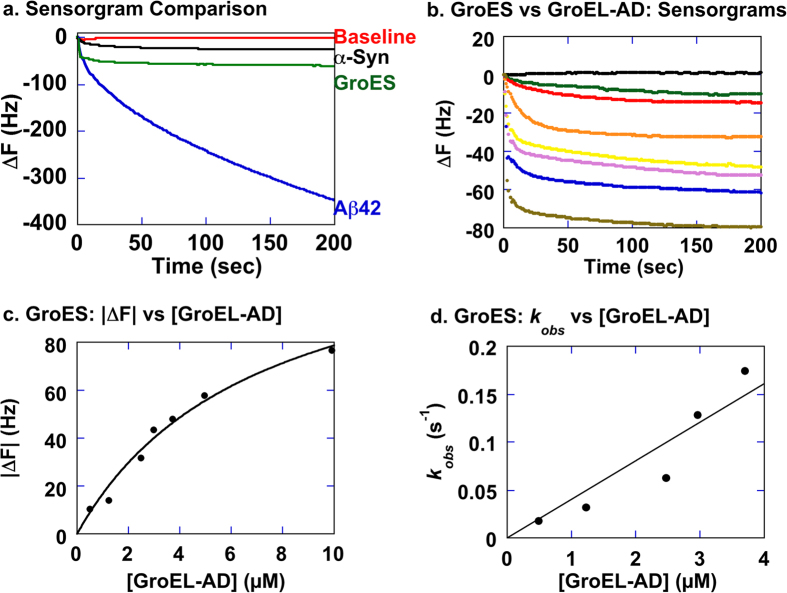
Binding interactions between GroEL-AD and various client proteins (α-Synuclein, Aβ42, and GroES) assessed by AffinixQNμ at 25 °C. (**a**) The concentration of protein used during immobilization to the quartz microbalance and the concentration of soluble protein added during subsequent measurements were both set to 100 ng/μl. The “Baseline” (*red*) denotes signal changes detected when buffer containing no protein is added to GroEL-AD immobilized sensors. The “α-Syn” (*black*) and “Aβ42” (*blue*) signals were measured by adding soluble aliquots of α-Synuclein or Aβ42, respectively, to the reaction chamber containing immobilized GroEL-AD. The “GroES” signal (*green*), however, was measured by adding soluble GroEL-AD to a reaction chamber containing immobilized GroES protein, in the presence of 0.4 M Gdn-HCl. See the Materials and Methods section for more details. (**b**) Sensorgrams measured using a quartz microbalance with immobilized GroES and varying concentrations of soluble GroEL-AD in the presence of 0.4 M Gdn-HCl. The concentration of GroEL-AD during each experiment was as follows (from top to bottom); 0 μM, 0.495 μM, 1.23 μM, 2.48 μM, 2.97 μM, 3.71 μM, 4.95 μM, 9.90 μM. Each trace was analyzed using the analysis function of Aqua 2.0 to obtain *k*_obs_ and ΔF values. (**c**) Plot of the estimated |ΔF| values to the concentration of soluble GroEL-AD added. Data points were fitted non-linearly to the isothermal adsorption equation outlined in Materials and Methods to obtain the fitted curve shown in the figure. (**d**) Linear regression plots of *k*_obs_ to the concentration of soluble [GroEL-AD]. We used only the *k*_obs_ values for the lower [GroEL-AD] concentrations in this analysis since the data sampling rate, which was fixed for the instrument, precluded the detailed sampling of raw sensorgrams with large *k*_obs_ values. This leads to more errors to be incorporated into the *k*_obs_ estimates at higher [GroEL-AD] concentrations, and subsequently a notable tendency in the linear regression analysis to yield negative values of *k*_off_ (the y-intercept).

**Figure 7:**
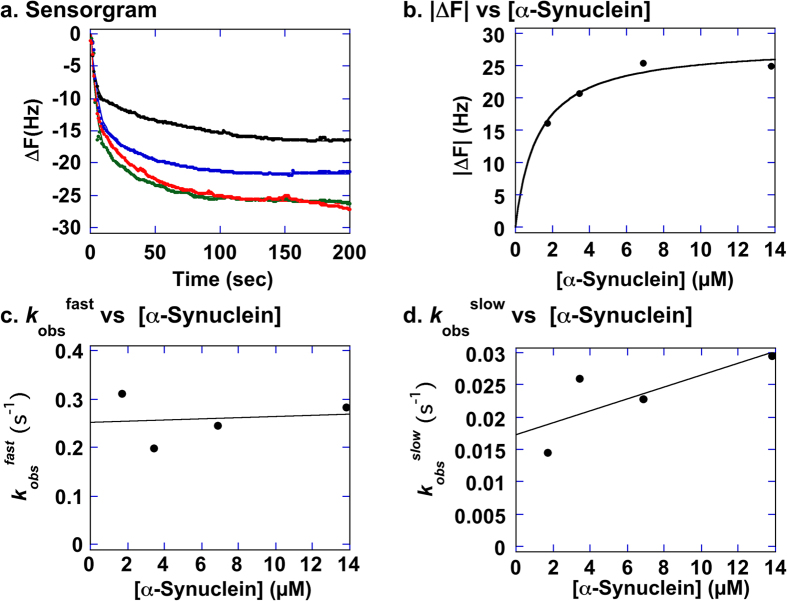
Analysis of α-Synuclein binding to immobilized GroEL-AD molecules using QCM. (**a**) Sensorgrams measured using a quartz microbalance with immobilized GroEL-AD and varying concentrations of soluble α-synuclein. The molar concentration of α-Synuclein used for each sensorgram was as follows from top to bottom: 1.72 μM, 3.45 μM, 6.90 μM (*green trace*), 13.8 μM (*red trace*). Raw data of each trace were fitted non-linearly to a two phase exponential decay equation to obtain two apparent amplitude values and two rate constants, *k*_obs_^fast^ and *k*_obs_^slow^. The net change in frequency, |ΔF|, was estimated by adding the two derived amplitudes of the analysis. (**b**) Non-linear fitting of |ΔF| values to the molar concentration of soluble GroEL-AD. See the Materials and Methods section for details on the analysis and the main text for derived *K*_d_ values. (**c**) Linear regression analysis of *k*_obs_^fast^ against [α-Synuclein]. (**d**) Linear regression analysis of *k*_obs_^slow^ against [α-Synuclein]. See main text for details and derived *K*_d_ values.

Analyses were performed using either the software package supplied by the manufacturer (Aqua 2.0; for single exponential decay w/drift; Fig. 6b), or KaleidaGraph 4.5.1 (all other analyses; Figs 6 and 7).

## Additional Information

**How to cite this article**: Ojha, B. *et al*. Suppression of amyloid fibrils using the GroEL apical domain. *Sci. Rep.* **6**, 31041; doi: 10.1038/srep31041 (2016).
